# Genetic differences between diagnosed and undiagnosed Celiac disease: a population-based study

**DOI:** 10.1007/s00439-025-02778-2

**Published:** 2025-09-29

**Authors:** Mohammad Sayeef Alam, Brooke N. Wolford, Kristian Hveem, Knut E. A. Lundin, Sebo Withoff, Iris H. Jonkers, Ludvig M. Sollid, Rebecka Hjort, Eivind Ness-Jensen

**Affiliations:** 1 HUNT Center for Molecular and Clinical Epidemiology, NTNU, Norwegian University of Science and Technology, Trondheim, Norway; 2 HUNT Research Centre, NTNU, Norwegian University of Science and Technology, Levanger, Norway; 3 Department of Gastroenterology, Oslo University Hospital - Rikshospitalet, Oslo, Norway; 4 Norwegian Coeliac Disease Research Centre, Institute of Clinical Medicine, University of Oslo, Oslo, Norway; 5 Department of Genetics, University Medical Center Groningen, University of Groningen, Groningen, Netherlands; 6 Department of Immunology, Oslo University Hospital - Rikshospitalet, Oslo, Norway; 7 Department of Medicine, Levanger Hospital, Nord-Trøndelag Hospital Trust, Levanger, Norway; 8 Department of Molecular Medicine and Surgery, Karolinska Institutet and Karolinska University Hospital, Stockholm, Sweden

## Abstract

A large proportion of individuals with celiac disease (CeD) remain undiagnosed, often presenting at an older age of onset or with non-classical symptoms compared to diagnosed cases. Such heterogeneity might be related to genetic factors. The aim was to utilize a CeD-screened adult population to compare the genetic variants in known and newly diagnosed cases. In the fourth wave of the population-based Trøndelag Health Study (HUNT4) 826 CeD and 51,516 non-CeD individuals were included. Medical registries identified 361 previously diagnosed cases, while screening identified 465 new cases. A validated polygenic risk score (PRS) was used to assess the genetic risk of CeD among the two case groups versus non-CeD individuals. Additional genetic variants not included in the PRS were also analyzed. The PRS distinguished cases from non-cases with high accuracy (AUROC: 85% for known cases, 83% for new cases). The genetic variation explained by the PRS was similar for known and new cases (17.1% versus 14.5%). The odds ratio for being in the highest genetic risk group (top 10%) was 22.7 (95% CI 14.1–36.4) for known cases and 18.6 (95% CI 12.4–27.9) for new cases versus the median group (40%-60%). Differences in effect size among specific genome-wide variants were observed but were not significantly associated with CeD. A validated PRS showed significant genetic difference between CeD cases and the general population, with similar association in both known and newly diagnosed cases. This suggests that genetic architectures of the two groups are comparable, implying that other non-genetic factors may drive CeD in adults.

**Supplementary Information:**

The online version contains supplementary material available at 10.1007/s00439-025-02778-2.

## Introduction

Celiac disease (CeD) is a chronic disorder due to an immune mediated reaction to gluten affecting the small intestine of genetically predisposed individuals (Ludvigsson 2013). The global prevalence of CeD is estimated to be between 1 and 2% (Makharia et al. 2022). Still, a large proportion of cases remains undiagnosed, likely because of heterogeneous symptoms and clinical presentations (Kang et al. 2013; Singh et al. 2018; Olano 2021). Hypothetically, this may be related to differences in genetic risk.

The genetic architecture of CeD is polygenic and complex. Each individual carries a unique combination of genetic variants, which determines the aggregated risk for the disease. The importance of human leukocyte antigen (HLA) allotypes and their role in the development of CeD are well defined (Sollid 2017). In addition, over 41 non-HLA loci with minor effects on CeD have been discovered through genome-wide association studies (GWASs) (van Heel et al. 2007; Dubois et al. 2010; Trynka et al. 2011; Ricaño-Ponce et al. 2020; Alam et al. 2025). These HLA and non-HLA SNPs together account for 30–40% of the heritability of CeD (Gutierrez-Achury et al. 2015).

We compared the distribution and risk associated with HLA-DQ allotypes between diagnosed and undiagnosed CeD but observed no clear differences (Hjort et al. 2024). However, genetic variants may exist in other HLA loci or in loci outside the HLA region, which remain to be investigated. To our knowledge, no previous study has compared the overall genetic makeup among known and new cases in a true population-based sample.

Hence, in the present study, the aim was to use a validated PRS to assess the genetic differences between CeD cases and non-cases and between previously known and newly diagnosed individuals detected through screening. In addition to the PRS, a genome-wide SNP-level comparison was done among the known and newly diagnosed individuals to explore any SNP specific effect differences.

## Methods

### Cohort

The study population was based on the fourth round of the Trøndelag Health Study (HUNT4), where all 103,800 adults (20 years or older) in Nord-Trøndelag County were invited between 2017 and 2019, of whom 56,042 (54%) participated. Comprehensive data on demographics, clinical measurements, and genetics were collected via questionnaires, interviews, clinical examinations, and biological samples with the help of health professionals. The HUNT study is a population-based health survey conducted at an interval of 11 years since the 1980ies to assess the changing health and well-being of the population (Åsvold et al. 2023).

### Celiac screening

All HUNT4 participants with available blood samples were first screened for CeD by a dual transglutaminase 2 (TG2) immunoglobulin (Ig) A and TG2 IgG assay (Klaasen et al. 2022). The seropositive participants (i.e., TG2-IgA ≥ 0.7 mg/L or TG2-IgG ≥ 1 mg/L) were then evaluated for histopathological signs via endoscopic biopsies. The biopsies were taken from the duodenum, specifically the duodenal pars horizontalis (D3) and the duodenal bulbus (D1). A confirmed diagnosis of CeD was established if signs corresponding to Marsh grade 3 showing villous atrophy were present according to the modified Marsh–Oberhuber classification (Oberhuber 2000) and other potential causes of inflammation, including NSAID use and *Helicobacter pylori *infections, were ruled out. The CeD screening followed the ESsCD guidelines detailed here (Al-Toma et al. 2019). Additionally, previous CeD cases were identified through linkages to the hospital records and Norwegian Patient Registry (using the unique birth number assigned to each individual) based on the ICD-10 diagnostic codes (K.90), procedure codes (UJD02, UJD05) and historical rate codes prior to 2006 (Lukina et al. 2024). HLA-typing was not used for screening or diagnosis of CeD (Lukina et al. 2024).

### Genotyping and quality control

Genotyping of blood samples of the HUNT participants has been performed via four different SNP arrays (HumanCoreExome12 v1.0, HumanCoreExome12 v1.1, UM HUNT Biobank v1.0, and UM HUNT Biobank v2.0). Approximately 358,964 genotyped SNPs remained after removing samples with a < 99% call rate and a Hardy–Weinberg deviation p value < 0.0001. Using the Positional Burrows Wheeler Transform (PBWT) method implemented in IMPUTE5 (Rubinacci et al. 2020) and Haplotype Reference Consortium (HRC) reference panel (McCarthy et al. 2016), approximately 24.9 million SNPs were imputed. Variants with an imputation accuracy of 0.3 or higher were retained for downstream GWAS analysis using SAIGE to account for case-control imbalance, large sample size and relatedness among the cohort (Zhou et al. 2018). The low threshold was set to enable a broader exploration of the SNP-level associations, rather than being limited to the smaller number of SNPs included in the PRS. Further details on the QC process are available elsewhere (Alam et al. 2025).

### Polygenic risk score (PRS)

The polygenic risk score (PRS) quantifies risk by evaluating the number of risk alleles each person carries. A previously validated PRS for CeD, comprising 1661 SNPs was reproduced and calculated as the sum of the number of risk alleles weighted by the effect size from the largest GWAS on CeD (Privé et al. 2022) using PLINK (Purcell et al. 2007). A penalized regression method was incorporated to determine the inclusion of SNPs in the PRS (Privé et al. 2022). SNPs (*n* = 79 [4.75%]) with poor imputation quality (r^2^ < 0.7) were excluded. Most of the excluded SNPs (*n* = 76 [96.2%]) were located in the HLA region; however, 56% of the total HLA coverage was still accounted for by the remaining SNPs. The PRS was rank inverse normalized before being used as a continuous exposure variable. It was also categorized into deciles. Apart from the overall PRS (PRS_all_), two more PRSs were created based on only the HLA SNPs (*n* = 58; PRS_HLA_) and only the non-HLA SNPs (*n* = 1524; PRS_non−HLA_), respectively.

### Statistical analysis and software

Nagelkerke’s R^2^ was used to estimate the proportion of variation explained by the PRSs. The discriminatory power of the PRS_all_ to distinguish high-genetic-risk individuals from the general population, i.e., between cases and non-cases, as well as between known and new cases, was assessed using the area under the receiver operating characteristic (AUROC) curve. Logistic regression was employed to estimate odds ratios (ORs) and 95% confidence intervals (CIs) for the association between CeD and each PRS_all_decile, using the 5th and 6th deciles as the reference category. All analyses and visualizations were performed in RStudio running R v4.1.2 or the Plink v1.9 tool on the Ubuntu 22.04.6 LTS operating system (Purcell et al. 2007; RStudio Team 2020; R Core Team 2021).

Additionally, separate genome-wide association analysis was conducted on the known and newly diagnosed individuals for CeD outcome. Based on the effect size and its range, only SNPs with the same directional OR and non-overlapping CIs between the known and new cases were recorded. The ANNOVAR (Wang et al. 2010) tool was used to annotate those variants and map them to the nearest gene. It was performed on hg19 build via the avsnp151 database. After ANNOVAR, the VarElect tool was used to perform disease/phenotype-dependent gene prioritization (Stelzer et al. 2016). VarElect leverages the extensive LifeMap Knowledgebase of GeneCards to prioritize the genes linked directly (explicitly mentioned in publication or associated with known disorders) or indirectly (via shared pathways and interactions with intermediate genes) on the phenotype (“coeliac” OR “celiac”). Z- and Q-test were also conducted to assess the differences and heterogeneity respectively among the observed OR of the known and new cases.

### Ethics and consent

The Regional Committee for Ethics in Medical Research Central approved the HUNT Study (#67445), the genotyping of the participants (#2014/144, #2018/1622, #152,023), and the present study (#7943). Written informed consent was received from all participants. The study was conducted in accordance with principles established by the Declaration of Helsinki.

## Results

### Study population

The study included 826 cases with CeD and 51,516 individuals without CeD. Among the cases, 361 had a previous diagnosis and 465 were new cases discovered during CeD screening. The median age at participation and inter-quartile range (IQR) of the total study population was 56 (42.3–68.7) years and 45% were men. Among the cases, the median age at participation for the known, previously diagnosed cases was 58 (42.4–68.7) years and 33% were men. The median age at participation for the newly diagnosed cases was 53 years (42.3–68.6) and 46% were men. The case identification steps are illustrated in a flowchart (Supplementary Fig. 1).

### Polygenic risk score distribution

**Fig. 1:**
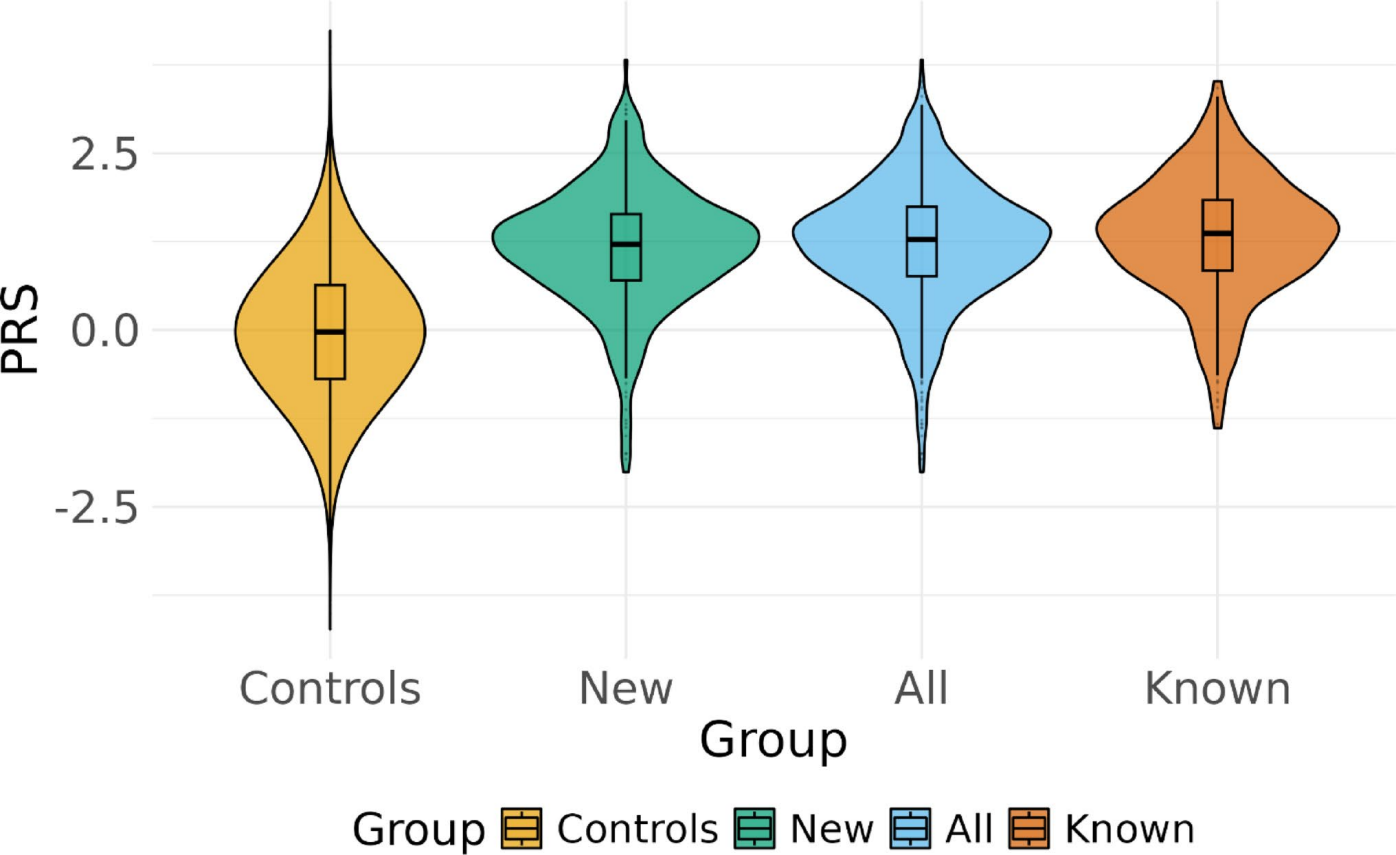
Violin Plot for Non-cases and CeD subgroups. The violin plot illustrates the distribution of the PRS_all_ among non-cases, all confirmed cases, known cases, and new cases. The bottom and top edges of the box represent the 25th (Q1) and 75th (Q3) percentiles, the line inside the box represents the median, and the whiskers show the range of the data within 1.5 times the interquartile range. The x-axis represents the PRS_all_, while the y-axis shows the density

The PRS_all_ was calculated with 1,582 SNPs, which provided 95.2% coverage of the original PRS. The distribution of the study population across PRS_all_ deciles is shown in Table 1, with the majority of the cases within the top decile. The median PRS_all_ was higher among cases compared to non-cases (Fig. 1). The distribution of PRS_HLA_ and PRS_non−HLA_ shows a similar pattern (Supplementary Fig. 2). Additionally, the chromosome-wise and within HLA, SNP imputation scores assured the inclusion of reliable variants (Supplementary Tables 1–2).

**Table 1:** Distribution of all non-cases and cases across PRS_all_ deciles

Decile	Non-cases *n* (%)	All cases *n* (%)	Known cases *n* (%)	New cases *n* (%)
1	5225 (10.1)	9 (1.1)	2 (0.6)	7 (1.5)
2	5226 (10.1)	8 (1)	4 (1.1)	4 (0.9)
3	5226 (10.1)	8 (1)	4 (1.1)	4 (0.9)
4	5212 (10.1)	22 (2.7)	11 (3)	11 (2.4)
5	5218 (10.1)	17 (2.1)	6 (1.7)	11 (2.4)
6	5206 (10.1)	28 (3.4)	13 (3.6)	15 (3.2)
7	5188 (10.1)	46 (5.6)	16 (4.4)	30 (6.5)
8	5144 (10)	90 (10.9)	32 (8.9)	58 (12.5)
9	5058 (9.8)	176 (21.3)	74 (20.5)	102 (21.9)
10	4813 (9.3)	422 (51.1)	199 (55.1)	223 (48)
Total	**51,516**	**826**	**361**	**465**

The values are presented as counts (n) and percentages (%)

* PRS* Polygenic risk score

### Performance

The total variability explained by the PRS_all_, including both HLA and non-HLA variants, was estimated at 17% for known cases and 14% for new cases. Out of these, the non-HLA SNPs variability explained 3.4% for known and 2.8% for new cases (Table 2).

**Table 2:** Comparison of variation explained (Nagelkerke’s R²) and predictive accuracy (AUROC) for PRS_all_, the PRS_HLA_, and the PRS_non-HLA_, across CeD subgroups

Group	PRS_all_ m, *n*^1^	PRS_HLA_ m, *n*^1^	PRS_non−HLA_ m, *n*^1^
All cases	0.17, 84.18	0.16, 83.82	0.03, 64.82
Known cases	0.17, 85.8	0.15, 85.06	0.03, 67.6
New cases	0.14, 83.61	0.14, 83.46	0.03, 64.97

*PRS* Polygenic risk score, *HLA* Human leukocyte antigen, *AUROC* Area under receiver operating characteristic curve

^1^ m: Nagelkerke’s R^2^ representing proportion of variation explained, n: AUROC is classification accuracy

**Fig. 2:**
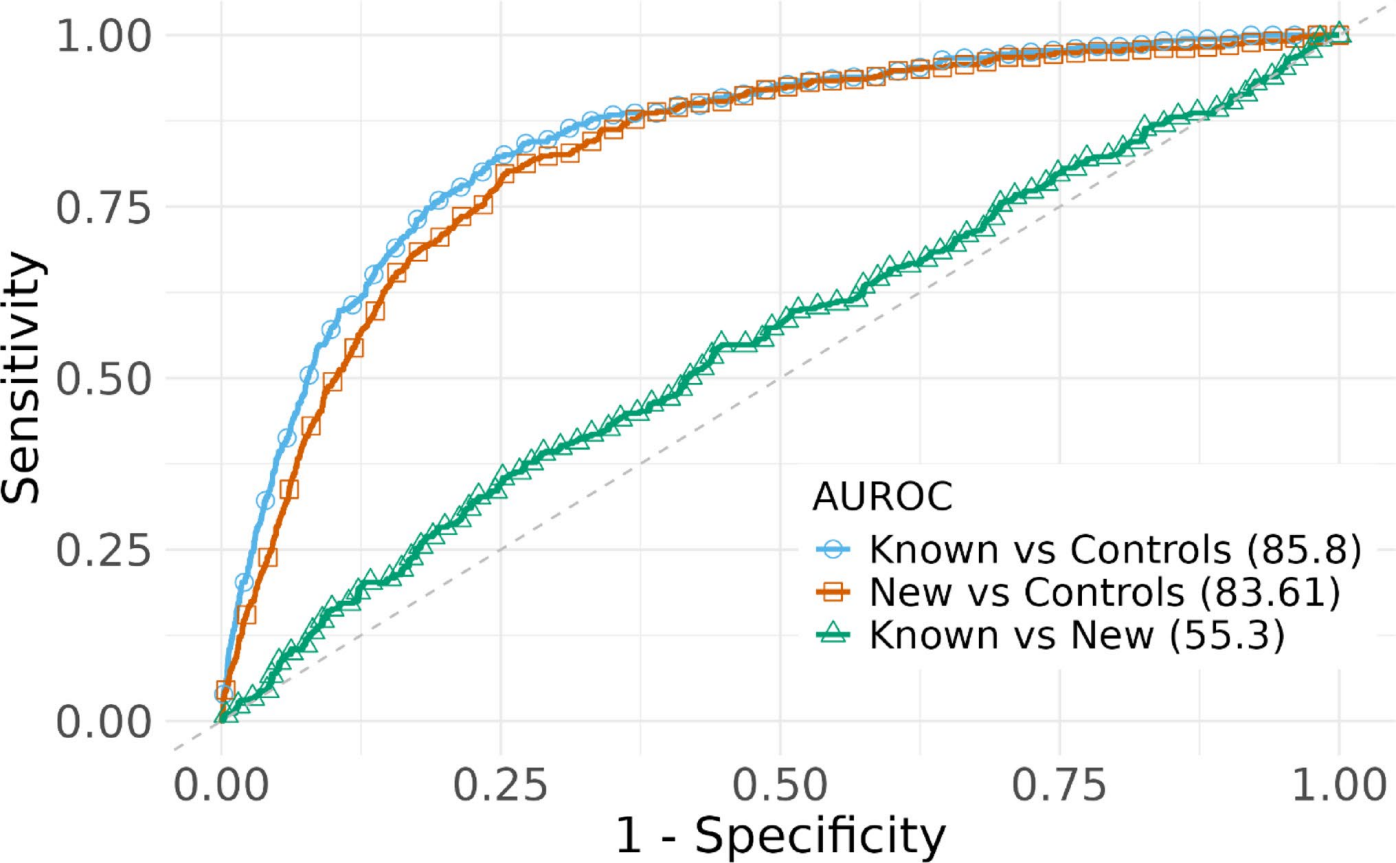
AUROC for the PRS_all_. The AUROC evaluates the performance of the PRS_all_ in distinguishing between non-cases and case subgroups. The x-axis represents the false positive rate, and the y-axis represents the true positive rate. *AUROC* Area Under the Receiver Operating Characteristic curve

The classification accuracy of the PRS_all_ showed high accuracy in distinguishing between cases and non-cases (up to 85%), but notably lower accuracy to differentiate between the case subgroups themselves (55%) (Fig. 2). The PRS_HLA_ classification accuracy was similar to that of the PRS_all_, with 85.06% and 83.46% AUROC for known and new cases respectively, whereas the accuracy for the PRS_non−HLA_ was estimated at 67.6% and 64.97%, respectively (Supplementary Fig. 3).

### Stratified associations

**Fig. 3:**
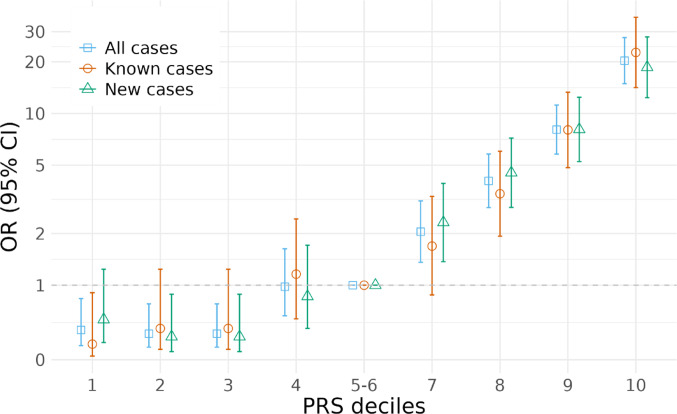
Odds Ratio of the PRS_all_ by Decile. The odds ratio (OR) and 95% confidence intervals (CI) of the PRS_all_ is plotted for each decile among all cases, known cases and new cases compared to non-cases

Individuals with higher PRS_all_ value tended to have higher odds of developing CeD. This increasing trend in OR was observed in both the known and new group compared to the reference population (Fig. 3). Having a PRS_all_ in the top decile was associated with an OR of 22.7 (95% CI 14.2–36.4) for known cases and 18.6 (95% CI 12.4–27.9) for new cases, compared to the population in the 40–60 percentile (Supplementary Table 3). The PRS_HLA_ showed a higher contribution to the overall association with CeD, particularly among individuals in the higher deciles, with ORs for the top decile estimated at 17.1 (95% CI 11-26.7) for known cases and 15.9 (95% CI 10.6–23.8) for new cases (Supplementary Table 4). The PRS_non−HLA_ exhibited a modest but consistent increase in odds across the deciles, with comparable estimates in CeD subgroups (Supplementary Table 5). The increasing trend in OR among the PRS_HLA_ and PRS_non−HLA_ is illustrated in a forest plot (Supplementary Fig. 4).

### Genome-wide comparison

To pinpoint which genes may contribute to each CeD subgroup, we conducted separate GWASs on both known and new cases. This identified 451 SNPs with the same directional ORs but varying effect sizes, as indicated by non-overlapping confidence intervals. Using ANNOVAR (Wang et al. 2010), the 451 SNPs were mapped to 199 genes. The difference among the ORs of the associated SNPs, ranged from 0.24 to 18.16 between known and new cases; however, none of the effect estimates reached genome-wide significance threshold (p-value < 5E-08).

The VarElect tool identified 19 genes with direct linkages, 133 genes with indirect linkages. Additionally, 40 and 7 genes that were labeled unrelated to CeD and unavailable in their LifeMap Knowledgebase respectively. Among the directly linked genes, 28 SNPs from five loci (*APP*, *CD226*, *DYRK1A*, *NR3C1*, and *SIRPA)* showed null effects in new cases (OR range: 1.02–1.11, 95% CI range: 0.84–1.46) but relatively larger effects in known cases (OR range: 1.40–2.12, 95% CI range: 1.21–2.93), with $\:P<9\times\:{10}^{-6}$. In addition, *APBB2*, *BICD1*, *CUL2*, *PAK5*, *PTPRG* genes were identified to have an indirect link with CeD through the previously established genes like *SH2B3*, *PTPN2*, *SOCS1*, *HLA-DQA1*, and *HLA-DQB1* among the newly diagnosed cases but not in the known cases. No statistically significant differences or heterogeneity were observed among the effect estimates of SNPs among the two case groups (Supplementary File 1).

## Discussion

As the present study focused on the genetic load rather than PRS development, we replicated the PRS from the largest and most comprehensive GWAS available (Privé et al. 2022) to ensure consistency with established summary statistics and prior research. The previously validated PRS was reproduced in the current cohort and was able to distinguish CeD cases from non-cases with high accuracy. Our findings confirmed previously observed gene-dose relationship (Romanos et al. 2014) in both previously known and newly diagnosed CeD cases. However, no significant differences were observed in the genetic architecture between the two subgroups.

A large proportion of genetic variability for CeD is explained by the *HLA-DQA1* and *HLA-DQB1* genes, of which the risk variants are necessary for CeD development. Consequently, the variability explained was greater among the PRS_HLA_ compared to PRS_non−HLA_. Furthermore, the PRS_non−HLA_ was not able to properly distinguish between cases and non-cases, and the PRS_all_showed negligible improvements after adding the non-HLA variants, which is in line with earlier reports (Romanos et al. 2014). To maximize the number of variants included in PRS_all_ while maintaining comparable accuracy, the 0.7 threshold was selected, as similar AUROC values were observed at both thresholds of 0.7 and 0.8. The removal of the low quality imputed HLA SNPs did not affect the classification accuracy. Since the PRS_all_ is not an additive function of the PRS_HLA_ and PRS_non−HLA_, the corresponding estimates should be interpreted with caution.

Large population-based studies comparing the genetic architecture of cases with a previous diagnosis to newly diagnosed cases are limited to our own study on HLA-DQ variants (Hjort et al. 2024). We speculated that potential differences would lie in other parts of the HLA or the non-HLA region. To the best of our knowledge, this is the first study comparing cases identified through hospital records to newly diagnosed cases, identified through screening, across the whole genome. However, there were no significant differences in variability explained or in risk estimates over PRS strata between known and new cases.

Notably, the original PRS (Privé et al. 2022) was based only on hospital-based cases, disregarding any SNPs potentially related only to the phenotype of new cases. Hence, the PRS_all_ may not be the best suited to capture differences between the subgroups. Future GWASs on CeD severity or nuances of symptoms may improve discrimination between new and known cases.

The genome-wide comparison identified SNPs outside the PRS_all_ which showed considerable effect estimates for some SNPs among new cases and null among known cases, and vice versa. However, heterogeneity was not observed upon empirical statistical testing. Moreover, none of the SNPs achieved genome-wide significance in our GWAS, and larger samples are required to explore associations further. It is also possible that unknown exogenic factors explain differences in disease severity and age at onset.

Major strengths of this study include CeD screening of the entire HUNT4 population and linkages with medical registries in Norway, which enabled inclusion of all cases, including previously undiagnosed individuals. The screening also minimized the misclassification bias common in population studies where undiagnosed or potential cases end up in the comparison group. Additionally, the relatively high response rate (54%) compared to similar large-scale studies (Schoeler et al. 2023), reduced the risk of selection bias. The new cases were all identified based on the same stringent criteria which are considered the gold standard for correct diagnosis. Still, as the known cases were identified based on diagnostic codes, misclassified individuals might be present in this case group.

Although the non-HLA SNPs were imputed at high quality (r^2^> 0.7), a limitation is the poor imputation of some of the HLA SNPs, which were excluded (~ 50%) from the original PRS. However, the remaining variants retained adequate accuracy, similar to the original study (Privé et al. 2022). This fact suggests that we managed to include the most important SNPs and that this did not significantly impact the results. However, we cannot rule out that this reduced the chance of finding true risk differences in known and new cases. The inclusion of all adults irrespective of age at diagnosis assured coverage of the whole population rather than limiting the study sample to specific high-risk groups or only to children. However, the absence of children and young individuals in the screening is a limitation along with the predominance of participants with genetic ancestry similar to individuals of European descent in HRC reference panel, which restricts the generalizability of the results.

To conclude, the PRS_all_ managed to significantly differentiate between cases and non-cases with marginal improvements when adding non-HLA variants. However, there were no significant genetic differences between known and previously undiagnosed cases. We show a PRS could help effectively segregate high-risk individuals from the general population for initial screening and earlier interventions. However, more refined PRSs are needed to distinguish between clinical subgroups of CeD, hence, the HLA-DQ typing followed by serology remains more feasible and equally effective for early detection. The lack of distinction between known and new cases in this study indicate that genetic risk alone does not explain disease severity or age at diagnosis, suggesting that environmental exposures acquired throughout adulthood are involved in CeD etiology for the new cases.

## Supplementary Information

Below is the link to the electronic supplementary material.

Supplementary Material 1

Supplementary Material 2

## Data Availability

The Trøndelag Health Study (HUNT) has invited individuals aged 13–100 years to four surveys between 1984 and 2019. Comprehensive data from more than 140,000 individuals having participated at least once and biological material from 78,000 individuals are collected. The data are stored in HUNT databank and biological material in HUNT biobank. HUNT Research Centre has permission from the Norwegian Data Inspectorate to store and handle these data. The key identification in the database is the personal identification number given to all Norwegians at birth or immigration, whilst de-identified data are sent to researchers upon approval of a research protocol by the Regional Ethical Committee and HUNT Research Centre. To protect participants’ privacy, HUNT Research Centre aims to limit storage of data outside HUNT databank and cannot deposit data in open repositories. HUNT databank has precise information on all data exported to different projects and are able to reproduce these on request. There are no restrictions regarding data export given approval of applications to HUNT Research Centre. For more information see: http://www.ntnu.edu/hunt/data.

## Code availability

The codes supporting the current study have not been deposited in a public repository because the pipeline is under construction but are available from the corresponding author on request.

## Declarations

### Web resources

GeneCards—https://www.genecards.org/. VarElect—https://varelect.genecards.org/about/. PGS Catalog—https://www.pgscatalog.org/.
