# Supplementary material for: Genetic differences between diagnosed and undiagnosed Celiac disease: a population-based study
Source: Hum Genet. 2025 Sep 29;144(11-12):1071–8. doi: 10.1007/s00439-025-02778-2 (PMC12689682; doi:10.1007/s00439-025-02778-2)
Supplement: Supplementary file 2 — Supplementary Material 2 [file 439_2025_2778_MOESM2_ESM.docx]

# **Supplementary Tables**

Supplementary Table 1: Imputation accuracy of all the SNPs in the PRS_all_ in HUNT4

| **Chromosome** | **Imputation accuracy** | | | | | | **Grand Total** |
| --- | --- | --- | --- | --- | --- | --- | --- |
|  | **<50** | **50-60** | **60-70** | **70-80** | **80-90** | **90-100** |  |
| **1** |  |  |  |  | 12 | 136 | **148** |
| **2** |  |  |  |  | 5 | 124 | **129** |
| **3** |  |  | 2 |  | 5 | 119 | **126** |
| **4** |  |  |  | 2 | 6 | 102 | **110** |
| **5** |  |  |  | 1 | 5 | 92 | **98** |
| **6** | 1 | 34 | 41 | 22 | 28 | 109 | **235** |
| **7** |  |  |  | 1 | 10 | 81 | **92** |
| **8** |  |  |  | 1 | 4 | 47 | **52** |
| **9** |  |  |  | 5 | 6 | 64 | **75** |
| **10** |  |  |  |  | 6 | 77 | **83** |
| **11** |  |  |  | 2 | 1 | 64 | **67** |
| **12** |  |  |  |  | 3 | 62 | **65** |
| **13** |  |  |  |  | 6 | 50 | **56** |
| **14** |  |  |  | 1 |  | 52 | **53** |
| **15** |  |  |  | 2 | 2 | 28 | **32** |
| **16** |  |  |  |  | 3 | 40 | **43** |
| **17** |  |  | 1 | 2 | 5 | 30 | **38** |
| **18** |  |  |  |  | 4 | 46 | **50** |
| **19** |  |  |  |  |  | 27 | **27** |
| **20** |  |  |  | 1 | 5 | 31 | **37** |
| **21** |  |  |  |  |  | 21 | **21** |
| **22** |  |  |  |  | 2 | 22 | **24** |
| **Grand Total** | **1** | **34** | **44** | **40** | **118** | **1424** | **1661** |

This table summarizes the imputation accuracy of all SNPs included in the PRS_all_, categorized by accuracy ranges (e.g., <50, 50–60, etc.). PRS – Polygenic risk score

Supplementary Table 2: Imputation accuracy of all the SNPs in the PRS_all_ in HUNT4 within the HLA region

| **Imputation accuracy** | **Count** |
| --- | --- |
| <50 | 1 |
| 50-60 | 34 |
| 60-70 | 41 |
| 70-80 | 21 |
| 80-90 | 21 |
| 90-100 | 16 |
| **Grand Total** | **134** |

This table provides the imputation accuracy of SNPs specifically within the HLA region, categorized by accuracy ranges. PRS – Polygenic risk score

Supplementary Table 3: Decile Odds Ratios for the PRS_all_ Among Case Subgroups

| **PRS_all_** | **Non-cases** | **All cases** | | **Known cases** | | **New cases** | |
| --- | --- | --- | --- | --- | --- | --- | --- |
| **Decile** | **n** | **n** | **OR (95% CI)** | **n** | **OR (95% CI)** | **n** | **OR (95% CI)** |
| **1** | 5225 | 9 | 0.4  (0.19-0.82)* | 2 | 0.21  (0.05-0.9)* | 7 | 0.54  (0.23-1.24) |
| **2** | 5226 | 8 | 0.35  (0.17-0.75)** | 4 | 0.42  (0.14-1.24) | 4 | 0.31  (0.11-0.88)* |
| **3** | 5226 | 8 | 0.35  (0.17-0.75)** | 4 | 0.42  (0.14-1.24) | 4 | 0.31  (0.11-0.88)* |
| **4** | 5212 | 22 | 0.98  (0.59-1.63) | 11 | 1.16  (0.55-2.43) | 11 | 0.85  (0.42-1.71) |
| **5-6** | 10424 | 45 | Reference | 19 | Reference | 26 | Reference |
| **7** | 5188 | 46 | 2.05  (1.36-3.1)*** | 16 | 1.69  (0.87-3.29) | 30 | 2.32  (1.37-3.92)** |
| **8** | 5144 | 90 | 4.05  (2.83-5.81)*** | 32 | 3.41  (1.93-6.03)*** | 58 | 4.52  (2.84-7.19)*** |
| **9** | 5058 | 176 | 8.06  (5.8-11.2)*** | 74 | 8.03  (4.84-13.3)*** | 102 | 8.08  (5.25-12.45)*** |
| **10** | 4813 | 422 | 20.31  (14.91-27.67)*** | 199 | 22.68  (14.15-36.36)*** | 223 | 18.58  (12.36-27.93)*** |

This table presents the odds ratios (OR) and 95% confidence intervals (CI) for each decile of the PRS_all_ among all cases, known cases, and new cases compared to non-cases. p < 0.05 (*), p < 0.01 (**), p < 0.001 (***).

Supplementary Table 4: Decile odds ratios (ORs) and 95% confidence intervals (CIs) among the different celiac disease subgroups compared to non-cases on the basis of the PRS_HLA_

| **PRS_HLA_** | **Non-cases** | **All cases** | | **Known cases** | | **New cases** | |
| --- | --- | --- | --- | --- | --- | --- | --- |
| **Decile** | **n** | **n** | **OR (95% CI)** | **n** | **OR (95% CI)** | **n** | **OR (95% CI)** |
| **1** | 5227 | 7 | 0.28  (0.13-0.63)** | 3 | 0.27  (0.08-0.91)* | 4 | 0.3  (0.1-0.84)* |
| **2** | 5222 | 12 | 0.49  (0.26-0.92)* | 3 | 0.27  (0.08-0.91)* | 9 | 0.67  (0.31-1.42) |
| **3** | 5225 | 9 | 0.37  (0.18-0.75)** | 4 | 0.36  (0.12-1.05). | 5 | 0.37  (0.14-0.96)* |
| **4** | 5221 | 13 | 0.53  (0.29-0.98)* | 7 | 0.64  (0.27-1.49) | 6 | 0.44  (0.18-1.07). |
| **5-6** | 10420 | 49 | Reference | 22 | Reference | 27 | Reference |
| **7** | 5181 | 53 | 2.18  (1.47-3.21)*** | 20 | 1.83  (1-3.35). | 33 | 2.46  (1.48-4.09)*** |
| **8** | 5154 | 80 | 3.3  (2.31-4.72)*** | 30 | 2.76  (1.59-4.78)*** | 50 | 3.74  (2.34-5.99)*** |
| **9** | 5006 | 227 | 9.64  (7.07-13.16)*** | 96 | 9.08  (5.71-14.45)*** | 131 | 10.1  (6.66-15.31)*** |
| **10** | 4860 | 376 | 16.45  (12.19-22.2)*** | 176 | 17.15  (11-26.75)*** | 200 | 15.88  (10.61-23.77)*** |

This table shows the odds ratios (OR) and 95% confidence intervals (CI) for each decile of the PRS_HLA_ among all cases, known cases, and new cases compared to non-cases. p < 0.05 (*), p < 0.01 (**), p < 0.001 (***). PRS – Polygenic risk score

Supplementary Table 5: Decile odds ratios among the different case subgroups on the basis of the PRS_non-HLA_

| **PRS_non-HLA_** | **Non-cases, n** | **All cases** | | **Known cases** | | **New cases** | |
| --- | --- | --- | --- | --- | --- | --- | --- |
| **Decile** |  | **n** | **OR (95% CI)** | **n** | **OR (95% CI)** | **n** | **OR (95% CI)** |
| **1** | 5191 | 43 | 0.57  (0.41-0.81)** | 15 | 0.44  (0.25-0.77)** | 28 | 0.69  (0.45-1.06). |
| **2** | 5192 | 42 | 0.56  (0.4-0.79)*** | 17 | 0.5  (0.29-0.85)* | 25 | 0.61  (0.39-0.96)* |
| **3** | 5177 | 57 | 0.76  (0.56-1.04). | 26 | 0.76  (0.48-1.2) | 31 | 0.76  (0.5-1.16) |
| **4** | 5169 | 65 | 0.87  (0.65-1.17) | 22 | 0.65  (0.4-1.05). | 43 | 1.06  (0.73-1.54) |
| **5-6** | 10320 | 149 | Reference | 68 | Reference | 81 | Reference |
| **7** | 5145 | 89 | 1.2  (0.92-1.56) | 37 | 1.09  (0.73-1.63) | 52 | 1.29  (0.91-1.83) |
| **8** | 5127 | 107 | 1.45  (1.13-1.86)** | 47 | 1.39  (0.96-2.02). | 60 | 1.49  (1.07-2.09)* |
| **9** | 5114 | 120 | 1.63  (1.27-2.07)*** | 54 | 1.6  (1.12-2.29)** | 66 | 1.64  (1.19-2.28)** |
| **10** | 5081 | 154 | 2.1  (1.67-2.64)*** | 75 | 2.24  (1.61-3.12)*** | 79 | 1.98  (1.45-2.71)*** |

This table provides the odds ratios (OR) and 95% confidence intervals (CI) for each decile of the PRS_non-HLA_ among all cases, known cases, and new cases compared to non-cases. p < 0.05 (*), p < 0.01 (**), p < 0.001 (***). PRS – Polygenic risk score

# **Supplementary Figures**


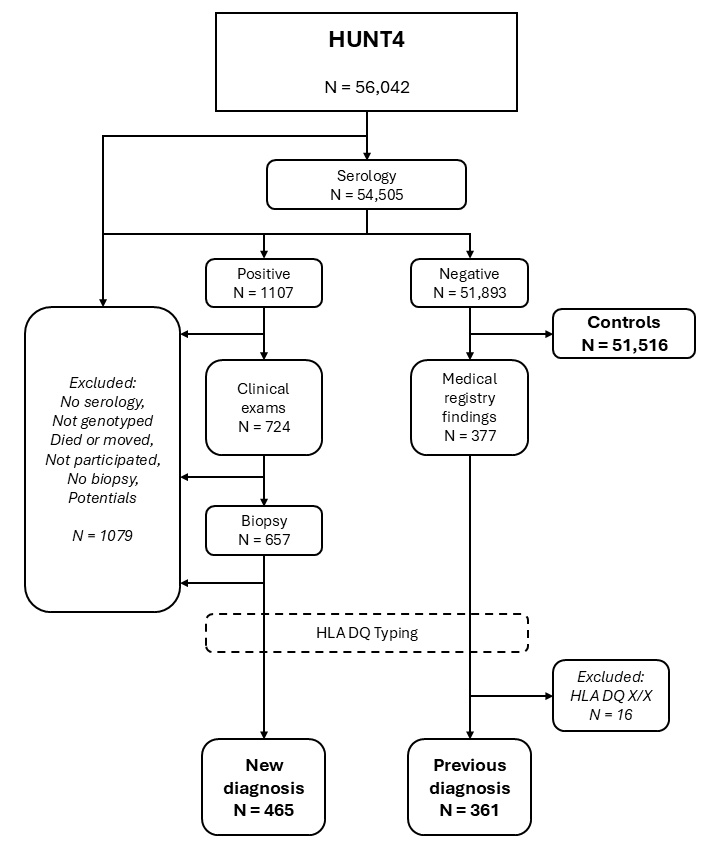


Supplementary Figure 1: Flowchart for Case Identification

This figure illustrates the process of identifying known and new cases from the screened population, including reasons for exclusion. The excluded box is split to show specific reasons for exclusion.


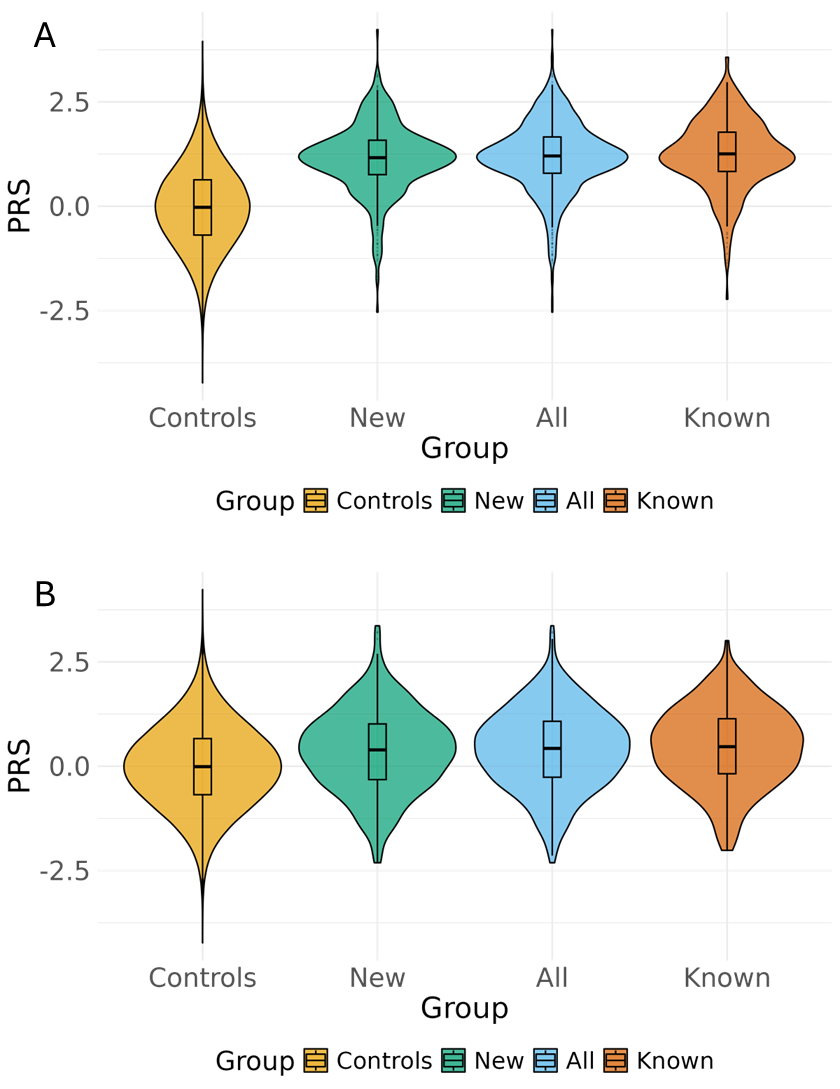


Supplementary Figure 2: Violin Plot of HLA and Non-HLA PRS Distribution

This violin plot A shows the distribution of) PRS_HLA_ among non-cases, all cases, known cases, and new cases. This violin plot B shows the distribution of) PRS_non-HLA_ among non-cases, all cases, known cases, and new cases. PRS – Polygenic risk score, HLA - Human Leukocyte Antigen


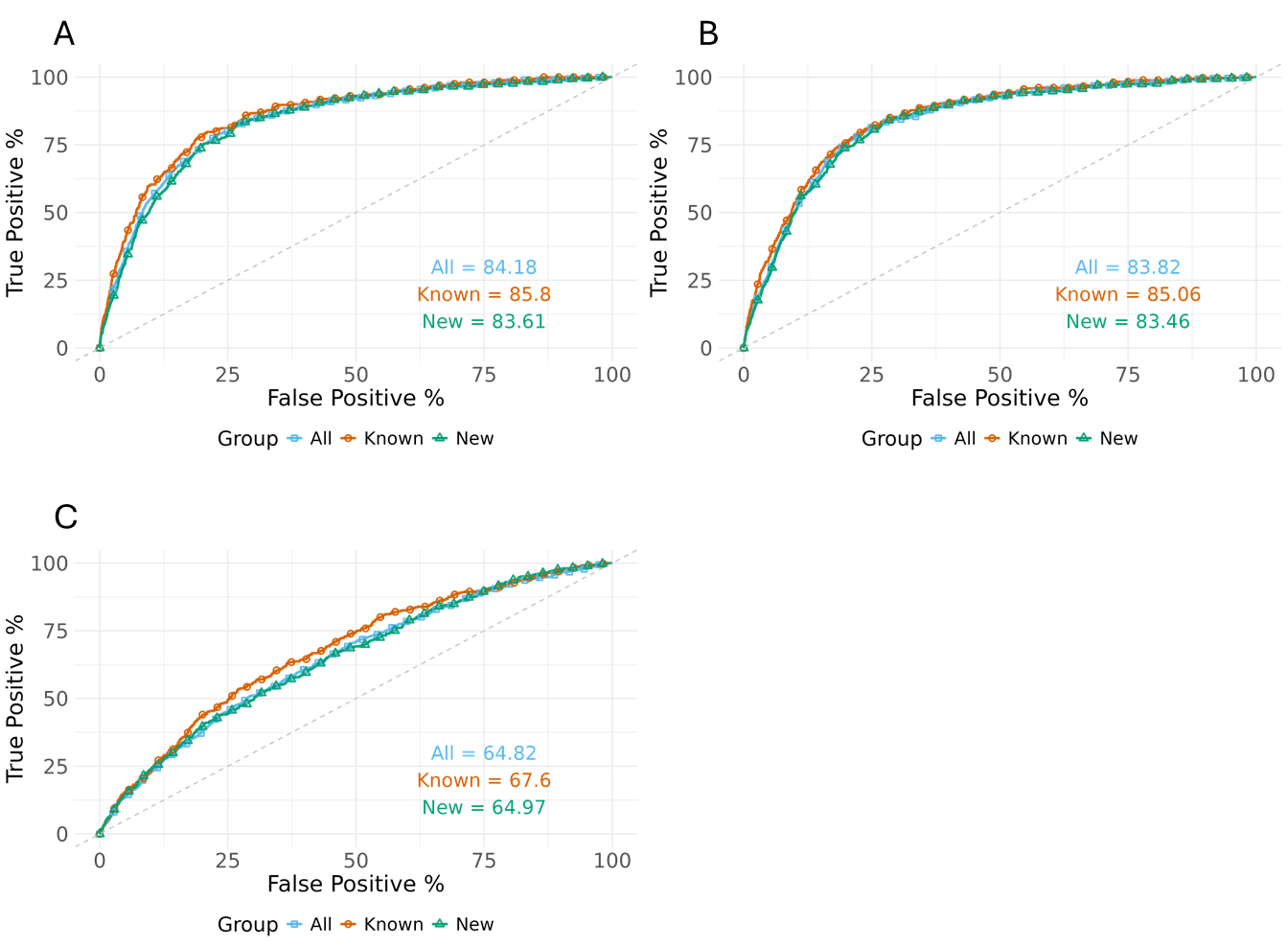


Supplementary Figure 3: AUROC for PRSs Among Case Subgroups

This figure A presents the AUROC (Area Under the Receiver Operating Characteristic curve) for the PRS_all_ in distinguishing between non-cases and case subgroups. The figure B shows the AUROC for the PRS_HLA_ in distinguishing between non-cases and case subgroups. This figure C shows the AUROC for the PRS_non-HLA_ in distinguishing between non-cases and case subgroups. PRS – Polygenic risk score


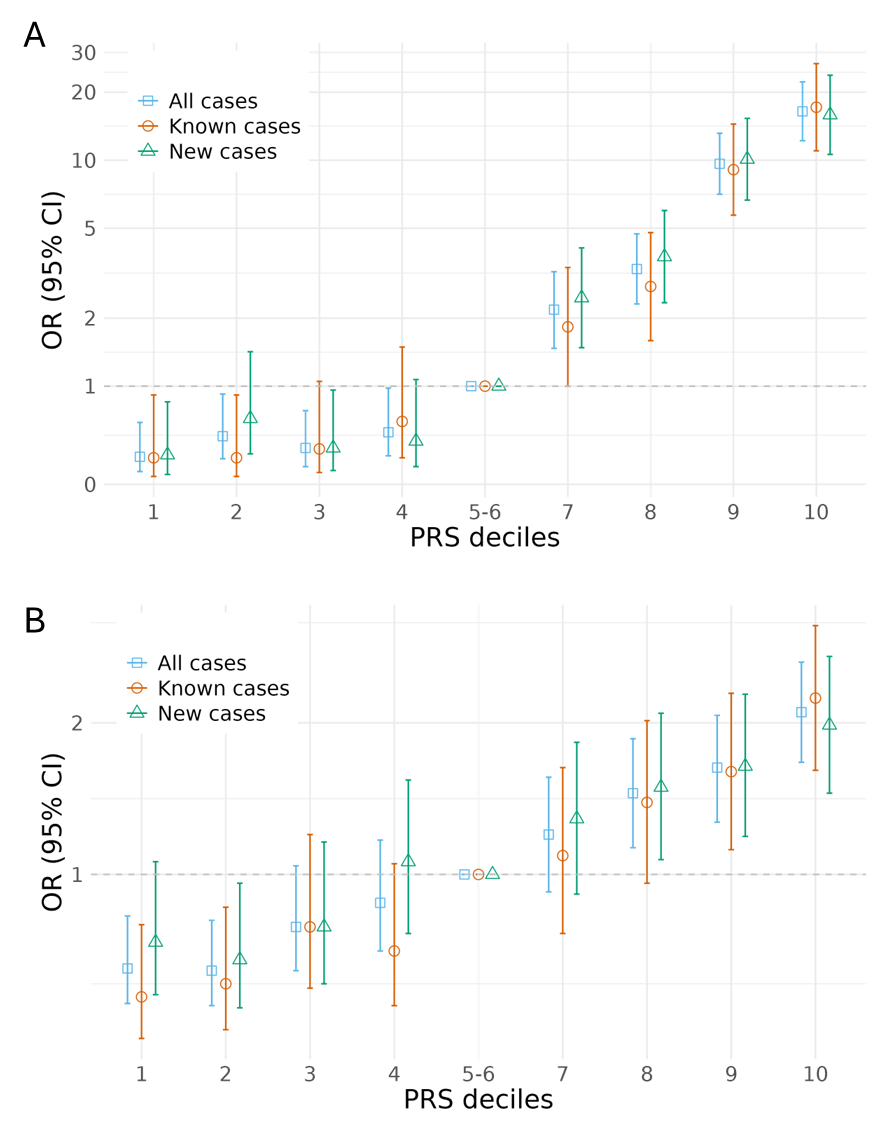


Supplementary Figure 4: Odds Ratio of the HLA and Non-HLA PRS by Decile

Forest plot in figure A illustrates the odds ratio of the PRS_HLA_ is plotted for each decile among different case subgroups compared to non-cases. Forest plot in figure B illustrates the odds ratio of the PRS_non-HLA_ is plotted for each decile among different case subgroups compared to non-cases. The x-axis represents the deciles, and the y-axis shows the odds ratio with 95% confidence intervals. PRS – Polygenic risk score
